# Molecular Detection and Genetic Diversity of Tick-Borne Pathogens in Goats from the Southern Part of Thailand

**DOI:** 10.3390/pathogens11040477

**Published:** 2022-04-15

**Authors:** Ruenruetai Udonsom, Aongart Mahittikorn, Charoonluk Jirapattharasate

**Affiliations:** 1 Department of Protozoology, Faculty of Tropical Medicine, Mahidol University, Bangkok 10400, Thailand; ruenruetai.udo@mahidol.ac.th (R.U.); aongart.mah@mahidol.edu (A.M.); 2 Department of Pre-Clinic and Applied Animal Science, Faculty of Veterinary Science, Mahidol University, Salaya, Nakhon Pathom 73170, Thailand

**Keywords:** *Anaplasma*, *Babesia*, *Theileria*, goats, phylogenetic tree, Thailand

## Abstract

Tick-borne hemoprotozoan and rickettsial diseases affect the health and productivity of small ruminants in tropical and subtropical regions. Despite the large population of goats in the southern part of Thailand, there is limited information on the prevalence of tick-borne pathogens. In this study, polymerase chain reaction was used to detect the presence of *Theileria* spp., *T. ovis*, *T. orientalis*, *Babesia ovis*, *Anaplasma ovis*, and *A. marginale* in 262 goats from three provinces in the southern part of Thailand. In this investigation, *Theileria* spp. and *A. ovis* were detected while *T. ovis*, *B. ovis*, and *A. marginale* were not detected. Overall infection rates of *Theileria* spp. and *A. ovis* were 10.3% and 1.5%, respectively. The co-infections of two parasites was observed in 1.5% of goats. Sequence analysis showed the presence of *T. luwenshuni* and *T. orientalis* in the goat samples. This study is the first to use the molecular detection of *T. orientalis* in Thai goats, and presents genetic characterization using the major piroplasm surface protein (MPSP) gene. In the phylogenetic analysis, the *T. orientalis* MPSP sequence was classified as type 7. The *A. ovis* major surface protein 4 (MSP4) gene sequences shared high identities and similarity with each other and clustered with isolates from other regions. This study provides information about the prevalence and genetic diversity of tick-borne pathogens in goats in the study area, and is expected to be valuable for the development of effective control measures to prevent disease in animals in Thailand.

## 1. Introduction

Tick-borne infections are one of the most common causes of decreases in livestock productivity, particularly in tropical and subtropical regions [1]. *Theileria* spp., *Babesia* spp. and *Anaplasma* spp. are important causative agents of tick-borne diseases in small ruminants, and have considerable economic impact [2,3].

Small ruminants are vulnerable to several species of *Theileria* including *Theileria lestoquardi*, *T. ovis*, *T. luwenshuni*, *T. uilenbergi*, *T. separata*, *T. recondita*, *Theileria* sp. OT3 and *Theileria* sp. MK [4,5,6]. Among these species, *T. lestoquardi*, *T. luwenshuni* and *T. uilenbergi* have been described as pathogenic, while *T. ovis*, *T. recondita* and *T. separata* cause benign theileriosis, a subclinical infection in goats and sheep [4,7,8]. *Babesia* species known to infect small ruminants include *Babesia ovis*, *B. motasi*, and *B. crassa* [9]. *B. ovis* and *B. motasi* are known to cause ovine babesiosis [9,10]. *B. ovis* is considered to be highly pathogenic in sheep, with mortality rates ranging from 30 to 50% [10].

*Anaplasma ovis* and *Anaplasma phagocytophilum* are rickettsial gram-negative organisms that cause ovine anaplasmosis. [11,12]. *A. ovis* is a major causative agent of ovine anaplasmosis. However, infection with *A. ovis* in sheep and goats is usually asymptomatic [12]. The infection is transmitted biologically and mechanically by ticks, blood-sucking insects, and contaminated blood [13].

In Thailand, goats are farmed primarily in the southern parts of the country. The goats are usually reared outdoors in mixed smallholder farms, and are therefore exposed to a range of vector-borne illness transmitted by arthropods such as ticks and blood-sucking insects. The prevalence of tick-borne pathogens (TBPs) has been investigated in various parts of Thailand [14,15,16,17], although these studies have focused on cattle and buffalo, and there is little information on infections in small ruminants. In the present study we investigated TBPs in goats from the southern part of Thailand, using polymerase chain reaction (PCR) assays. We also examined the prevalence and genetic diversity of the pathogens in goats. The phylogenetic relationships among the isolates identified in this research and those isolated from different countries were assessed.

## 2. Results

### 2.1. PCR Detection of TBPs in Goat Samples

In the present study, none of the 262 blood samples obtained were positive for *B. bovis*, *T. ovis*, or *A. marginale*. *Theileria* spp. and *A. ovis* were detected in the goat populations (Table 1). It was found that 13.3% (35/262) of goats from the southern part of Thailand were positive for at least one pathogen. The infection rates of TBPs in Yala, Pattani, and Narathiwat provinces were 4.9% (13/262), 4.1% (11/262) and 2.3% (6/262). *Theileria* spp. (10.3%, 27/262) were the most prevalent single infection, followed by *A. ovis* (1.5%, 4/262). Co-infections with *Theileria* spp. and *A. ovis* were found in 1.5% (4/262) of samples.

### 2.2. Sequencing Analysis

**Figure 1:**
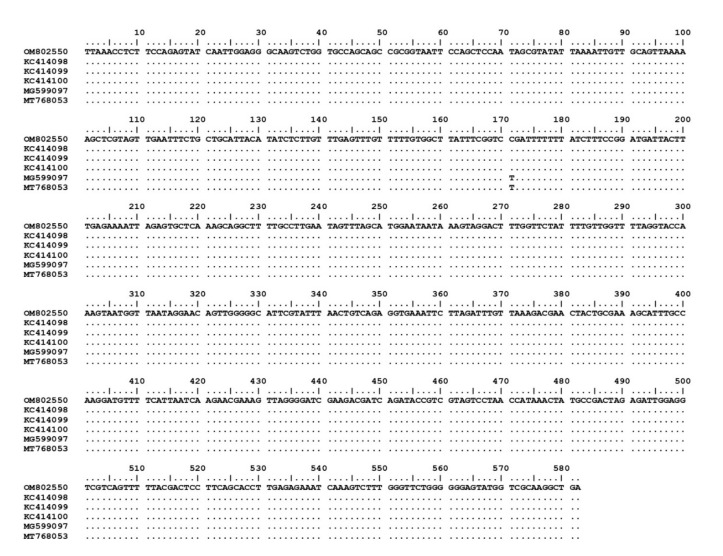
Nucleotide sequence alignment results of *T. orientalis* 18S rRNA gene isolate from goat in this study (OM802550) and database sequences.

**Figure 2:**
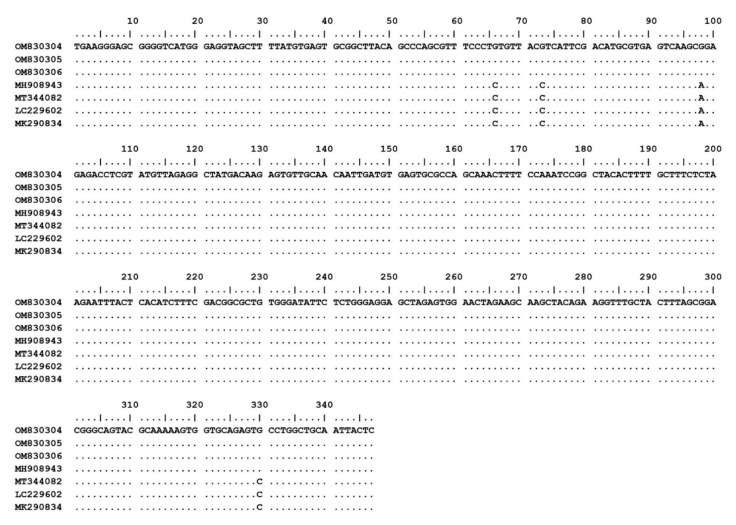
Nucleotide sequence alignment results of MSP4 gene of *A. ovis* isolates from goats (OM830304–OM830306) in this study and database sequences.

The 15 *Theileria* spp. isolates (OM802536–OM802550) were sequenced and the sequences compared with each other and with previously published sequences. Fourteen of the isolates (OM802536–OM802549) had 99.3–100% similarity to *T. luwenshuni* isolates from goats in Thailand (MW307318, MW307319 and MW307320) and sheep in China (KC414093 and KC414097), and another (OM802550) revealed 99.8–100% similarity to *T. orientalis* isolates from sheep in China (KC414098, KC 414099 and KC414100), cattle in Pakistan (MG599097) and ticks in India (MT768053) (Figure 1). The *T. orientalis* MPSP gene sequences had 99.6–100% similarity to *T. orientalis* MPSP gene isolates from various countries (AB871321, AB871336, AB562533, LC125433, and KU356867). The nucleotide sequence identity value of *A. ovis* MSP4 in this study (OM830304–OM830306) was 100% among the isolated sequences and 98.8–99.1% with previously published sequences (MH908943, MT344082, LC229602 and MK290834) (Figure 2).

### 2.3. Phylogenetic Analysis

**Figure 3:**
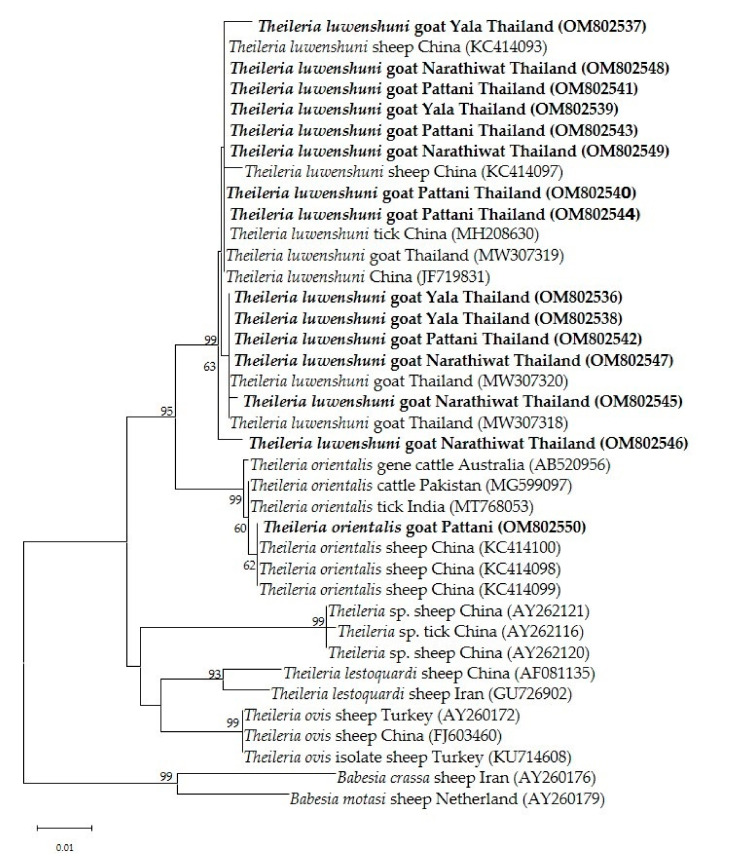
The phylogenetic tree of 18S rRNA gene partial sequences of *T. luwenshuni* and *T. orientalis* in this study (boldface letters) and representative *Theileria* species. The tree was constructed using maximum likelihood with the Kimura-2 method with 1000 bootstrap replications. Bootstrap values are indicated at each node and the numbers over 60 are shown in the tree. *B. crassa* and *B. motasi* 18S rRNA gene partial sequences were used as outgroups.

**Figure 4:**
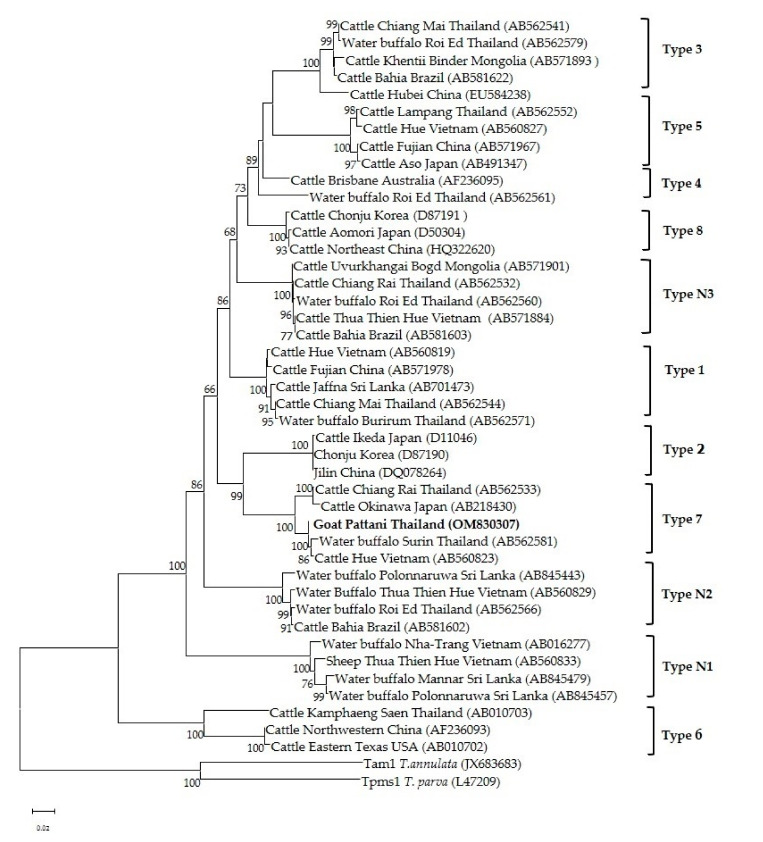
The phylogenetic tree of *T. orientalis* MPSP gene sequence in this study (boldface letters) and from global isolates. The tree was constructed using the neighbor-joining method with 1000 bootstrap replications. Bootstrap values are indicated at each node and the numbers over 60 are shown in the tree. The Tams1 gene of *T. annulate* and *T. parva* were used as outgroups.

Phylogenetic analysis of the *Theileria* spp. 18s rRNA gene revealed that *T. luwenshuni* was present in the three provinces of the southern part of Thailand. In the phylogenetic tree, the 14 sequences from Yala, Pattani and Narathiwat formed a cluster with the *T. luwenshuni* gene sequences previously isolated from goats in Thailand and sheep in China (Figure 3). One isolate clustered with *T. orientalis* isolated from sheep in China and cattle from Australia and Pakistan (Figure 3). One *T. orientalis* MPSP gene sequence in this study was located in the type 7 clade, and was closely related to isolates reported in cattle from Thailand, Japan and Vietnam (Figure 4).

**Figure 5:**
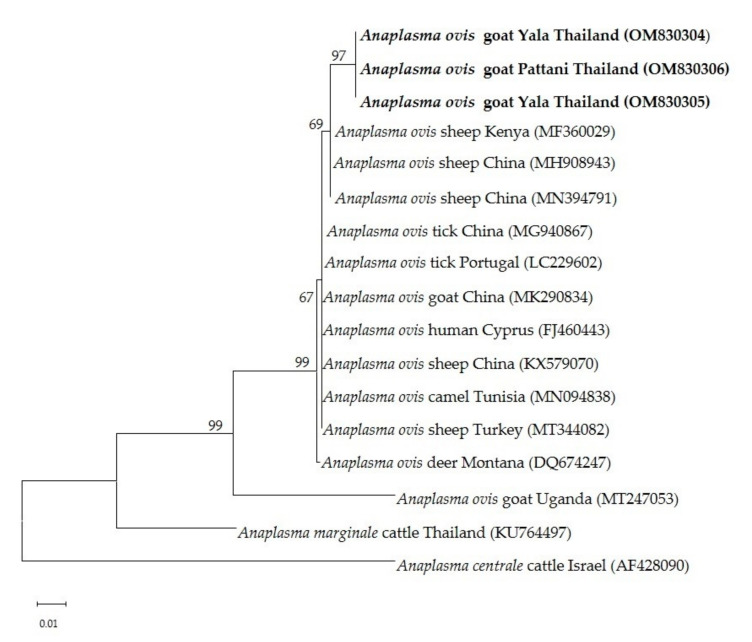
Phylogenetic relationship of *A. ovis* MSP4 sequences from goats in this study (boldface letters) and reference sequences from Genbank database. The tree was constructed using maximum likelihood with Kimura-2 method with 1000 bootstrap replications. Bootstrap values are indicated at each node and the numbers over 60 are shown in the tree. The sequences of *A. marginale* and *A. centrale* MSP4 gene were used as outgroups.

The phylogenetic tree showed that the *A. ovis* MSP4 genes from three isolates were confined to the same clade and had a close relationship with sequences from goats and sheep in China, Turkey and Kenya (Figure 5).

## 3. Discussion

Ovine tick-borne pathogen infections affect goats and sheep worldwide, and are widely distributed in tropical and subtropical areas [18]. In Southeast Asia, the occurrence of TBPs in goat and sheep has been investigated in countries including China [19,20,21], South Korea [22], Vietnam [23] and Myanmar [24]. However, little information on their prevalence and distribution in Thailand is available. In the present study, the prevalence and genetic characteristics of *A. ovis* and *Theileria* spp. infections of goats in three provinces from the southern part of Thailand were investigated.

Ovine anaplasmosis is a tick-borne disease caused by *A. ovis*, and affects goats and sheep worldwide [11]. The parasite is believed to induce asymptomatic infection in small ruminants [12], but cases of clinical illness have been reported, with symptoms including anaemia, pale mucous membranes, weight loss, abortion and death [25]. Little research has been done in Thailand into the prevalence of *Anaplasma* spp. infection in small ruminants. In this study, *A. ovis* was detected in 1.5% (4/262) of the goat samples. The prevalence of infection with the parasite was lower than previously reported in goats from Sa Kaeo province, Thailand (3.6%) [26], China (15.3–63.8%) [11,21,27], Bangladesh (14.7%) [28], Pakistan (16.6%) [29] and South Korea (17.9%) [22]. Several factors might have contributed to the differences in rates of infection with *A. ovis*, including vector activity, climate, variation in the susceptibilities of animals from different geographical areas and sample sizes. Further studies into vectors and seasonal dynamics, and the use of a larger number of samples from different provinces will be valuable for the more precise determination of the presence of *A. ovis* in Thailand.

The MSP4 gene of *Anaplasma* spp. is widely used for genetic characterization [30]. The sequences of the MSP4 genes obtained in the current study were highly conserved among the goat isolates, and were identical to previous isolates from China [31], Tur-key [32] and Portugal [33]. Phylogenetic analysis revealed that all of the *A. ovis* MSP4 sequences clustered into one clade, together with sequences from China, Kenya, Portugal, Cyprus, Tunisia and Turkey, suggesting that the *A. ovis* isolates in the study belong to one genotype. These findings are consistent with those of a previous study that reported that low or high genetic diversity of the MSP4 gene is associated with low or high prevalence of *A. ovis* [34,35]. The MSP4 gene in *A. ovis* evolves relatively rapidly, and has produced many pseudogenes [36]. Previous studies have identified two genotypes of *A. ovis* MSP4 from Uganda and four from China [27,37].

Theileriosis caused by *Theileria* spp is an important tick-borne disease of domestic animals and causes economic losses in many countries [38]. In this study, the overall prevalence of *Theileria* spp. was 10.3%, a proportion which is lower than the previously detected 41.3% in goats from Sakaeo province, Thailand [39]. In the neighbouring countries, the prevalence rates of *Theileria* spp. in goats was 34.7–52.7% from China [19,40,41], 33.3% from Myanmar [24], 8.5% from Bangladesh [42] and 13.4% from Uganda [37].

The 18S small subunit ribosomal RNA (18S rRNA) gene is widely used in the detection of *Theileria* spp., and this gene has been successfully used to distinguish the previously unknown *Theileria* spp. [19]. Sequence analysis revealed the presence of *T. luwenshuni* and *T. orientalis* in this study. The partial *T. luwenshuni* 18s rRNA gene sequence of the goat isolates from the southern part of Thailand had 99.3–100% nucleotide sequence identity with those of *T. luwenshuni* from Rayong province in Thailand and from strains isolated in China. A previous study reported a prevalence of 5% of *T. luwenshuni* in the Rayong and Chonburi provinces of Thailand [43]. *T. luwenshuni* is widely distributed in the small ruminant population in China [19,21,31,41], but its distribution in Thai goats is unknown. The results of the phylogenetic analysis revealed that the *T. luwenshuni* isolates in this research were closely related to *T. luwenshuni* isolates from China, suggesting that the parasite may have invaded Thailand through the passage of infected tick vectors or animals from China. Further epidemiological studies, taking into account mechanical or biological vectors for the transmission of *T. luwenshuni* and risk factor analysis, will be necessary to understand the current distribution of the pathogen in small ruminants in Thailand.

The partial *T. orientalis* 18s rRNA gene sequence of the goat isolate had 99.8–100% nucleotide sequence identity with those of the China, Pakistan and India isolates. *T. orientalis* has been reported to have a high prevalence in cattle and buffalo in Thailand [14,15,16]. This finding could indicate that the pathogen is in circulation among the animal farms in this country. To the best of the authors’ knowledge, this is the first report of the detection of *T. orientalis* in Thai goats. This agent has been detected in sheep blood samples from China [19] and Vietnam [14]. *Haemaphysalis* spp. are biological vectors for *T. orientalis* [44]. However, these tick species are not frequently found on livestock in Thailand, which are mainly infested with *Rhipicephalus microplus* [45]. The DNA of *T. orientalis* has been detected in the *R. microplus* [14]; however, it is unclear whether this tick may act as a mechanical or biological vector for this parasite. The biting arthropods are considered to be potential vectors for the transmission of *Theileria* spp. [46,47]. Further studies into tick vectors and biting arthropods will be needed to clarify the transmission of *T. orientalis* in Thailand.

The MPSP gene has been highly useful as a marker for investigating the phylogeny of *T. orientalis*, and is also used for diagnostic purposes [14,15,16,48]. *T. orientalis* isolates from different countries have been classified into 11 types (types 1–8 and types N1–N3) based on the MPSP gene sequences [48]. Phylogenetic analysis in this study revealed that the *T. orientalis* MPSP sequence of the goat isolate was of type 7 and formed a cluster with *T. orientalis* gene sequences previously isolated from cattle in Thailand, Vietnam and Japan. Previous research on genetic diversity of *T. orientalis* in cattle in Thailand categorized MPSP gene isolates into five types (types 1, 3, 5, 7 and N3) [14,15,16]. However, in the current study we were unable to detect other MPSP types due to the limited number of positive samples. Therefore, a large-scale study with a much larger sample size from different provinces should be performed.

In this study, *B. ovis*, *T. ovis* and *A. marginale* were not detected in goat samples, suggesting that these parasites are not common in the study area. However, *T. ovis* was detected in biting flies (*Tabanus megalops*) in Nakhon Pathom province, Thailand [47]. Further studies are required to determine whether these parasites are present in small ruminants in Thailand.

In conclusion, we detected the presence of *A. ovis* and *Theileria* spp. in domestic goats from the southern part of Thailand. The prevalence of the parasites was lower than those reported in several other countries. Sequence analysis showed that at least two species of *Theileria* currently exist in Thai goats. Here we describe for the first time the detection of *T. orientalis* in goat samples. The phylogenetic analysis revealed one *T. orientalis* MPSP type and *A. ovis* MSP4 type. Our results improve the understanding of the epidemiology of TBPs in the goat population in Thailand. However, continuous surveillance and updating of the occurrence of TBPs in small ruminants in this country should be undertaken.

## 4. Materials and Methods

### 4.1. Ethical Statement

The blood samples were obtained from a project aimed at the evaluation of *Toxoplasma gondii* and *Neospora caninum* specific recombinant proteins for the diagnosis of Toxoplasmosis and Neosporosis in cattle and goats. Use of goat blood samples in this study was approved by the Ethics and Animal Care and Use Committee of the Faculty of Veterinary Science, Mahidol University (Permit Number: MUVS-2021-12-53).

### 4.2. Animal Samples and DNA Extraction

**Figure 6:**
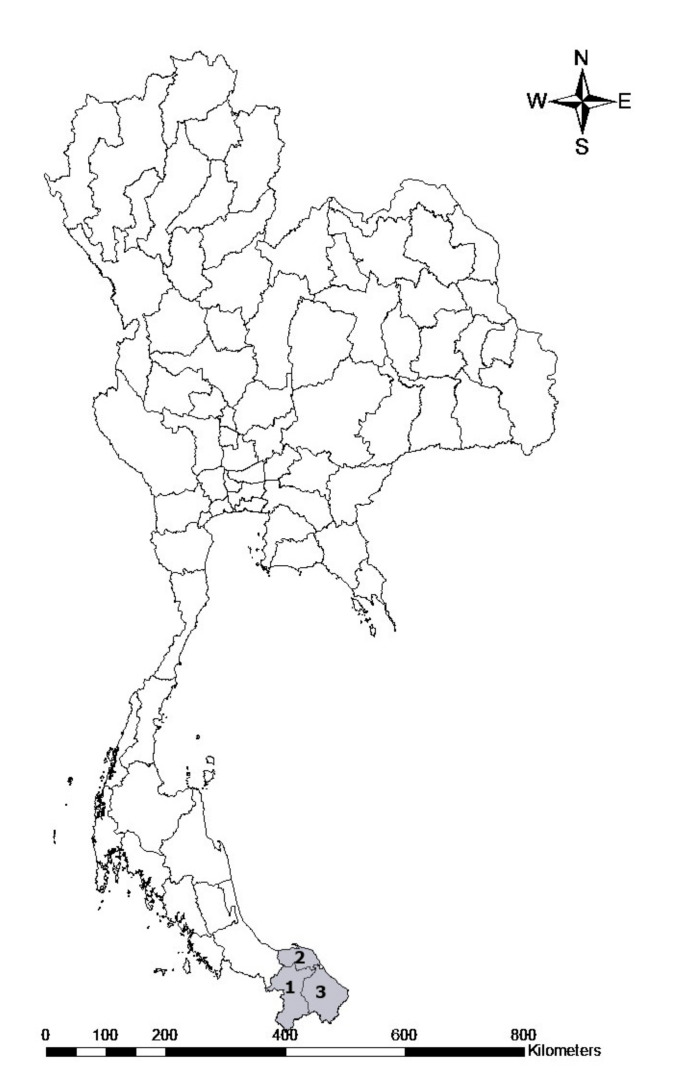
Map of sampling areas in the Sothern part of Thailand. The numbers represent the provinces where the goat samples were collected: 1. Yala, 2. Pattani, and 3. Narathiwat.

A total of 262 blood samples were collected from randomly selected herds from three provinces in the southern part of Thailand, including the Yala, Pattani and Narathiwat provinces (Figure 6). These blood samples were collected between March and April, 2021. The animals were restrained and blood was obtained from the jugular vein and transferred into 10 mL blood collection tubes containing anticoagulant. The samples were carried to the laboratory in a cooled box with ice packs and kept at 4 °C until they were used.

A total of 200 mL of blood sample was used to extract and purify genomic DNA using G-spin™ Total DNA extraction kits (iNtRON Biotechnology, Inc., Seongnam, South Korea) according to the manufacturer’s instructions. The extracted DNA was stored at −20 °C until they were used.

### 4.3. Molecular Detection of TBPs and DNA Sequencing

PCR assays were performed using primers for specific genes of *Theileria* spp., *T. orientalis*, *T. ovis*, *B. ovis*, *A. ovis* and *A. marginale*, as previously described [19,49,50,51,52]. Parasite detection was based on procedures previously described, as shown in Table 2.

A final volume of 25 µL was composed of 5 µL of 5X One *Taq* standard reaction buffer, 0.5 µL of deoxyribonucleotide triphosphates (dNTPs), 0.2 µM of each primer, and 0.125 µL of One *Taq* DNA polymerase (New England Biolabs, Ipswich, MA, USA), and topped up with distilled water to the final volume. DNA samples previously confirmed to be positive for *Theileria* spp., *A. ovis* and *A. marginale* were used as positive controls, whereas double-distilled water was used as the negative control. The positive controls of *B. ovis* and *T. ovis* were not available in our laboratory, so the correct size of PCR product was confirmed after sequencing. The PCR products were separated by gel electrophoresis on 1.5% agarose in 1 × TAE buffer and visualised using FluoroDye™ DNA Fluorescent Loading Dye (SMOBIO Technology, Hsinchu City, Taiwan) under a UV transilluminator.

Positive amplicons were snipped and extracted from agarose gel using Nucleo-Spin^®^ Gel and PCR Clean-up (Macherey-Nagel, Düren, Germany). The extracts concentration was measured using a NanoDrop 2000 spectrophotometer (Thermo Fisher Scientific, Waltham, MA, USA). The purified product was submitted for Sanger DNA sequencing (Macrogen, Seoul, Korea). Bioedit version 7.2.6 (Tom Hall Ibis Biosciences, CA, USA) was used to analyze the nucleotide sequences, and GenBank BLASTn analysis was used to evaluate their identities and similarities [53]. The percent identities between nucleotides were computed by pairwise distances using MEGA version X software (www.megasoftware.net, accessed on 3 March 2022) [54].

### 4.4. Phylogenetic Analysis

The pathogen sequences in this study were compared to sequences from other regions of the world banked in genetic databases using MEGA version X software [53]. Multiple sequence alignment was conducted using the Muscle programme for each locus, and the genetic relatedness was determined using neighbour-joining or maximum likelihood. The confidence in the branching pattern of the trees was estimated using bootstrap tests with 1000 replications.

## Figures and Tables

**Table 1:** The PCR screening results for *Theileria* spp. ana *A. ovis* infections in goats the from the southern region.

Province	No. of Tested Goats	*A. ovis* (%)	*Theileria* spp. (%)	Mixed Infections (%)	Isolated Number of *T. luwenshuni*	Isolated Number of *T. orientalis*
Yala	100	3 (1.1)	11 (4.2)	2 (0.7)	4	0
Pattani	100	1(0.4)	10 (3.8)	1 * (0.4)	5	1
Narathiwat	62	0	6 (2.3)	1 * (0.4)	5	0
Total	262	4 (1.5)	27 (10.3)	4 (1.5)	14	1

* Co-infections between *T. luwenshuni* and *A. ovis*.

**Table 2:** List of target genes and primers used for PCR assays.

Target Gene	Assay	Primer Sequences (5′→3′)		Fragment (bp)	Annealing Temp (°C)	Reference
		Forward	Reverse			
*A. ovis* (MSP4)	PCR	TGAAGGGAGCGGGGTCATGGG	GAGTAATTGCAGCCAGGCACTCT	347	62	[49]
*A. marginale* (MSP4)	PCR	CTGAAGGGGGAGTAATGGG	GGTAATAGCTGCCAGAGATTCC	344	60	[49]
*B. ovis* (18S rRNA)	PCR	TGGGCAGGACCTTGGTTCTTCT	CCGCGTAGCGCCGGCTAAATA	549	62	[50]
*T. ovis* (18S rRNA)	PCR	TCGAGACCTTCGGGT	TCCGGACATTGTAAAACAAA	520	60	[51]
*T. orientalis* (MPSP)	PCR	CTTTGCCTAGGATACTTCCT	ACGGCAAGTGGTGAGAACT	776	58	[52]
*Theileria* spp. (18S rRNA)	PCR nPCR	GAAACGGCTACCACATCT TTAAACCTCTTCCAGAGT	AGTTTCCCCGTGTTGAGT TCAGCCTTGCGACCATAC	778 581	55 55	[19]
